# *IL-1ɑ C376A* Transversion Variant and Risk of Idiopathic Male Infertility in Iranian Men: A Genetic Association Study

**DOI:** 10.22074/ijfs.2018.5375

**Published:** 2018-06-20

**Authors:** Tayyebeh Zamani-Badi, Mohammad Karimian, Abolfazl Azami Tameh, Hossein Nikzad

**Affiliations:** 1 Gametogenesis Research Center, Kashan University of Medical Sciences, Kashan, Iran; 2 Anatomical Sciences Research Center, Kashan University of Medical Sciences, Kashan, Iran

**Keywords:** Genetic Polymorphism, Interleukin-1α, Male Infertility, Spermatogenesis

## Abstract

**Background:**

*IL-1α* produced by Sertoli cells is considered to act as a growth factor for spermatogonia. In this study, we investigated the association of the C376A polymorphism in *IL-1α* with male infertility in men referring to the Kashan IVF Center.

**Materials and Methods:**

In this case-control study, 2 ml of blood was collected from 230 fertile and 230 infertile men. After DNA extraction, the C376A variant was genotyped by polymerase chain reaction-restriction fragment length polymorphism (PCR-RFLP). In addition, the molecular effects of the C376A transversion were analysed using bioinformatics tools.

**Results:**

A significant association was observed between the homozygous genotype CC with male infertility [odds ratio (OR)=1.97, 95% confidence interval (CI)=1.14-3.41, P=0.016)]. Carriers of C (AC+CC) showed a similar risk for male infertility (OR=1.78, 95% CI=1.06-2.99, P=0.030). Also, allelic analysis showed that the C allele is associated with male infertility (OR=1.43, 95% CI=1.09-1.88, P=0.011). In sub-group analysis, we found that the AC genotype is associated with asthenozoospermia (OR=2.38, 95% CI=1.03-5.53, P=0.043). In addition, carriers of C were at high risk for asthenozoospermia (OR=2.25, 95% CI=1.01-4.10, P=0.047). Also, C allele was significantly associated with oligozoospermia (OR=1.44, 95% CI=1.01-2.06, P=0.049) and non-obstructive azoospermia (OR=1.67, 95% CI =1.04-2.68, P=0.034). Finally, in silico analysis showed that the C376A polymorphism could alter splicing especially in the acceptor site.

**Conclusion:**

This is the preliminary report on the association of *IL-1α C376A* polymorphism with male infertility in the Kashan population. This association shows that the *IL-1α* gene may be a biomarker for male infertility, and therefore needs additional investigations in future studies to validate this.

## Introduction

Male infertility is a multifactorial syndrome that affects up to 12% of men (1). Male factors are responsible for 40-50% of total infertility cases (2). In more than 70% of cases, there is a conclusive reason including varicocele, aneuploidies, infectious diseases and post-testicular obstruction, however, in less than 30% of infertile males, the cause of their infertility is unknown and are thus diagnosed as idiopathic (3, 4). 

Environmental, lifestyle, physiological and genetic factors are involved in male infertility (5-7). From numerous genetic factors that are essential for normal spermatogenesis, cytokines play an important role (8). These are regulatory peptides which regulate testicular and glandular function (9).

Human seminal plasma contains several cytokines in. cluding IL-1, IL-2, IL-4, IL-5, IL-6, IL-7, IL-10, IL-11, IL-12, IL-13, IL-17, IL-18, IL-23, TNFa, IFN-., TGFa, TGFß (8). One of the most important gene sets involved in fertility is the interleukin-1 (*IL-1*) gene family which encodes regulatory cytokines playing multifaceted roles in the male reproductive system. For example, they may act as growth factors and are involved in physiological protection, germ cell proliferation and differentiation, regulation of junctions and steroidogenesis (10, 11). 

The *IL-1* gene family members include *IL-1α*(OMIM: 147760),IL-1ß(OMIM: 147720) and IL-1RA(OMIM: 147679), all of which are located on chromosome 2q14 (12). *IL-1α* is secreted from seminiferous epithelium and is known as a growth factor for immature Sertoli cells and spermatogonia (13). 

Single nucleotide polymorphisms (SNPs), by altering the structure of genes involved in spermatogenesis, may affect gene expression, mRNA structure and protein function, and may therefore lead to male infertility (14-16). Therefore, evaluating SNPs in the *IL-1* gene family could be considered as an interesting research topic. A SNP (C376A; rs2071376) has been found to have a high frequency in the *IL-1α* gene. The association of this SNP with some disorders has been investigated in different studies including cancers (17, 18), systemic sclerosis (19), periodontitis (20), endometriosis (21) and keratoconus (22). The association between the C376A SNP and idiopathic male infertility has, however, not been reported. In this study, we investigated the association between the *IL-1α* C376A SNP and idiopathic male infertility in an Iranian population as a preliminary project. Also, we evaluated the functional effects of C376A on *IL-1α* using bioinformatics tools.

## Materials and Methods

### Subjects and inclusion criteria

In this cross-sectional study, a total of 460 samples comprising 230 infertile men (with mean age of 30.93 ± 5.47) and 230 fertile men (with mean age of 32.12 ± 5.52) selected among individuals attending the Kashan Infertility Centre (Shahid Beheshti Hospital, Kashan, Iran). Infertile patients were defined as ‘idiopathic’ and selected based on andrological examination. Patients with previous testis trauma, obstruction of the vas deferens, infectious and chronic diseases, hypogonadotropic hypogonadism, abnormal hormonal profile (Luteinizing, Follicle Stimulating, and testosterone hormones) and abnormal karyotype or Y chromosome microdeletions were excluded from the study. According to the World Health Organization (WHO) 1999 criteria, the patient sub-groups were determined (23) and the subjects were categorized into non-obstructive azoospermia (n=51) without spermatozoa in the ejaculated semen, oligozoospermia (n=95) with sperm concentration less than 20 million/ml, and asthenozoospermia (n=84) with progressive sperm motility less than 50%. 

The control group was randomly selected from healthly men referred to the Kashan Infertility Centre. They had normal sperm parameters, had no history of chronic and familial diseases and had at least one offspring. Finally, a total of 2 ml of whole blood was collected from all males into EDTA-K3 containing tubes and were stored in -20°C for further usage. Written informed consent was obtained from all case and control subjects. The study was approved by the Medical Research Ethics Committee of the Kashan University of Medical Sciences (IR.KAUMS.REC.1394.6).

### Single nucleotide polymorphism genotyping

Total genomic DNA was isolated from whole blood by using a DNA extraction kit (Bioneer, Korea). Purified DNA was stored at -20°C for further use. The *IL-1α* C376A SNP was genotyped by the polymerase chain reaction- restriction fragment length polymorphism (PCR- RFLP) method. For this purpose, forward and reverse primers flanking the SNP were designed based on the complete sequence of *IL-1α* by the Oligo7 software (Molecular Biology Insights, Inc., Cascade, CO, USA).

The sequences of the primers were:

5´-ATGCTAAAATTACCGTGATTCT-3´ 

5´-AGATCAATGGAATAAATGGATG-3´ respectively.

The PCR was carried out in a total volume of 20 µl containing 10µl pre-mix (CinnaGen, Iran), 0.35 µM of each forward and reverse primers, and 3 µl of template DNA. PCR cycling conditions were an initial denaturation step at 94°C for 5 minutes followed by 35 cycles of denaturation at 94°C for 45 seconds, annealing at 56.9°C for 1 minute and extension at 72°C for 1 minute along with a final extension at 72°C for 5 minutes. PCR products were then digested with the *BstYI* restriction enzyme (CinnaGen, Iran). For this purpose, approximately 0.1 µg of the PCR product was incubated with 5 units of *BstYI* at 37°C for 16 hours. Finally, *BstYI* was inactivated by incubation at 65°C for 20 minutes. The digested fragments were separated on a 1% agarose gel stained with DNA Green Viewer (CinnaGen, Iran) and visualised under the UV light. To verify PCR-RFLP results, 2% of samples were sequenced randomly. PCR product recovery kit (Roche Applied Science, Mannheim, Germany) was used to purify the PCR product (368 bp in length). Direct sequencing of the purified PCR products was undertaken by Bioneer (Daejeon, Korea). Chromas (version 2.33) was used to check the chromatograms.

### Statistical analysis 

The difference in frequencies of genotypes and alleles between the case and control groups was analyzed by Chi-square test. For association analysis, the odds ratios (ORs) and 95% confidence intervals (95% CI) were estimated by a binary regression logistic test. A two-tailed p- value less than 0.05 (P<0.05) was considered significant. All analyses were conducted in the SPSS software (SSPS Inc., IBM Corp, Armonk, NY, USA) version 19.

### In silico analysis

Bioinformatics tools were used to analyze the influence of the *IL-1α* C376A intronic SNP on RNA structure and splicing pattern. The effect on RNA structure and splicing was assessed with RNAsnp online server (24) and NetGene2 (25) respectively. Finally, reported interactions of *IL-1α* with other molecules were obtained from the BioGRID interactome database (26).

## Results

### Polymerase chain reaction-restriction fragment length polymorphism and DNA sequencing

**Fig.1:**
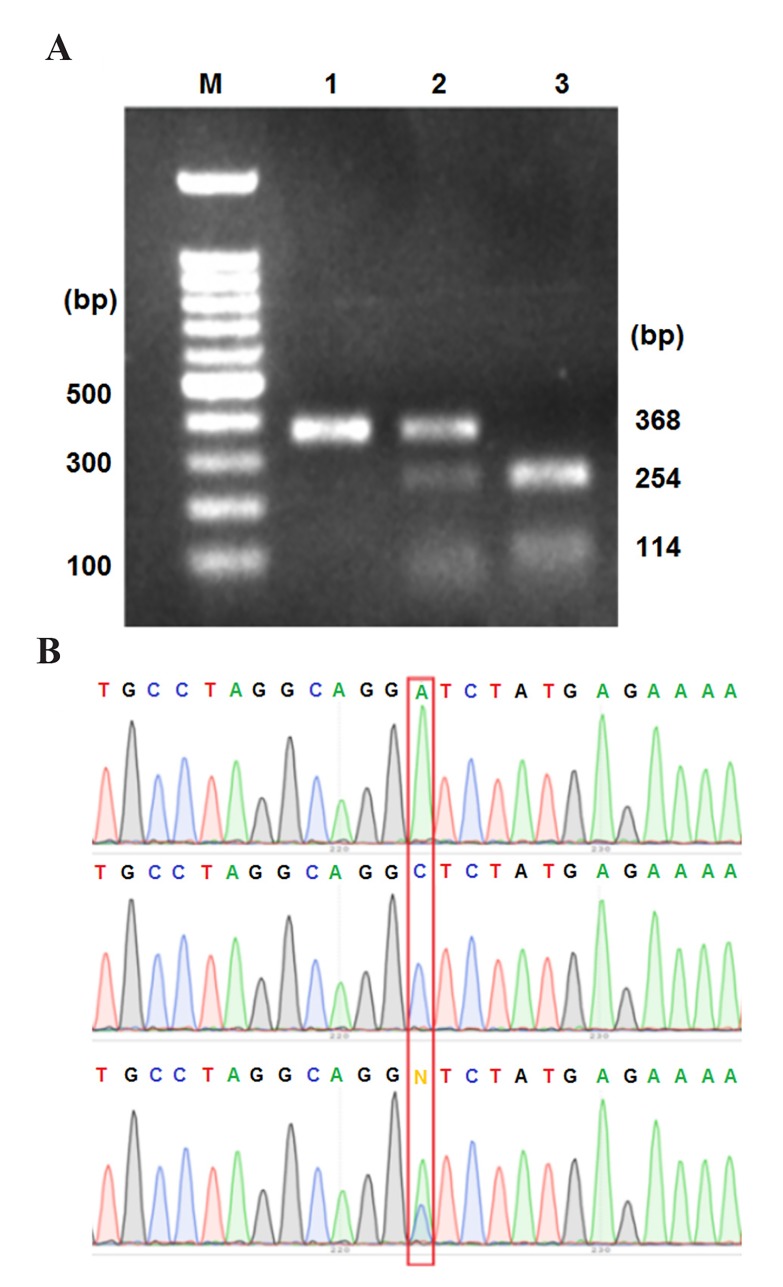
Polymerase chain reaction-restriction fragment length polymorphism (PCR-RFLP) and DNA sequencing results. A. The M, 1, 2 and 3 lanes show the 100 bp DNA ladder, and the AA, AC and CC genotypes, respectively and B. Partial sequence of IL-1. flanking the single nucleotide polymorphism (SNP) (red box).

Results of PCR-RFLP showed that 368 bp fragment was fully digested into 114 bp and 254 bp fragments in some samples, showing the efficiency of the method used. The samples with two, three and one fragments were identified as CC, AC, and AA genotypes respectively (Fig .1A). The data from direct sequencing also confirmed the results of PCR-RFLP (Fig .1B). 

### *IL-1α* C376A distribution 

In this study, the genotype and allele frequencies of the *IL-1α* C376A SNP were compared between the infertile and healthy groups (Table 1). We observed a significant association between the homozygous genotype CC with male infertility (OR=1.97, 95% CI=1.14-3.41, P=0.016). Carriers of C (AC+CC) were at a similar risk for male infertility (OR=1.78, 95% CI=1.06-2.99, P=0.030). Also, allelic analysis showed that the C allele is associated with infertility (OR=1.43, 95% CI=1.09-1.88, P=0.011). In sub-group analysis, we found that the AC genotype is associated with asthenozoospermia (OR=2.38, 95% CI=1.03-5.53, P=0.043). In addition, there was a significant association between carriers of C and asthenozoospermia (OR=2.25, 95% CI=1.01-4.10, P=0.047). Also, C allele was significantly associated with oligozoospermia (OR=1.44, 95% CI=1.01-2.06, P=0.049) and non-obstructive azoospermia (OR=1.67, 95% CI=1.04-2.68, P=0.034).

### In silico analysis

**Fig.2:**
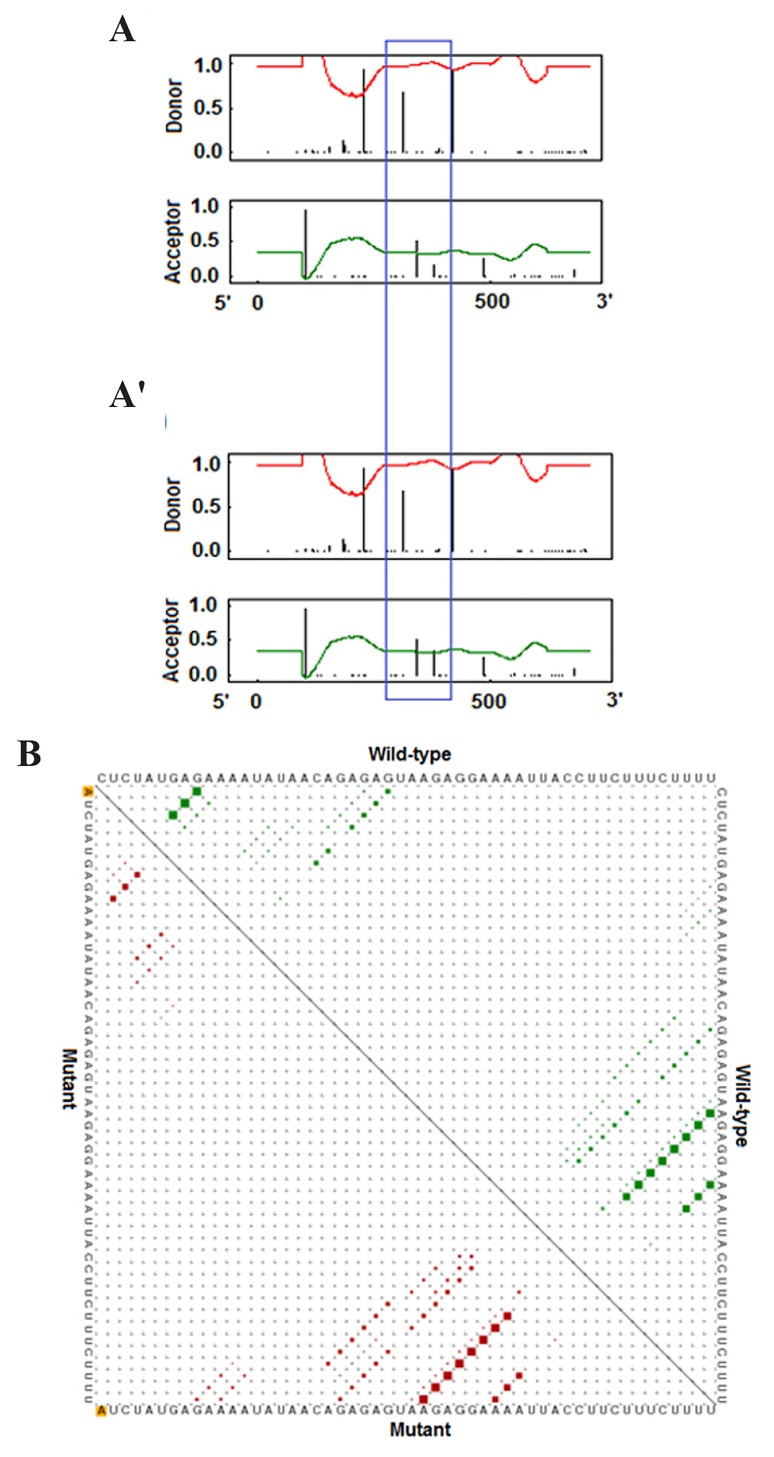
Results of NetGene2 and RNAsnp. A. Splice sites prediction by NetGene2 when nucleotide A is present at the C376A position, A’. Splice sites pattern after the C substitution at the C376A position. Some changes were observed after the substitution especially in the acceptor site (the differences between the splice patterns are shown by the blue box), and B. The presumptions of variant and ancestral sequences are introduced in lower and upper triangle of the plots respectively. The single nucleotide polymorphism (SNP) is highlighted by yellow color.

**Fig.3:**
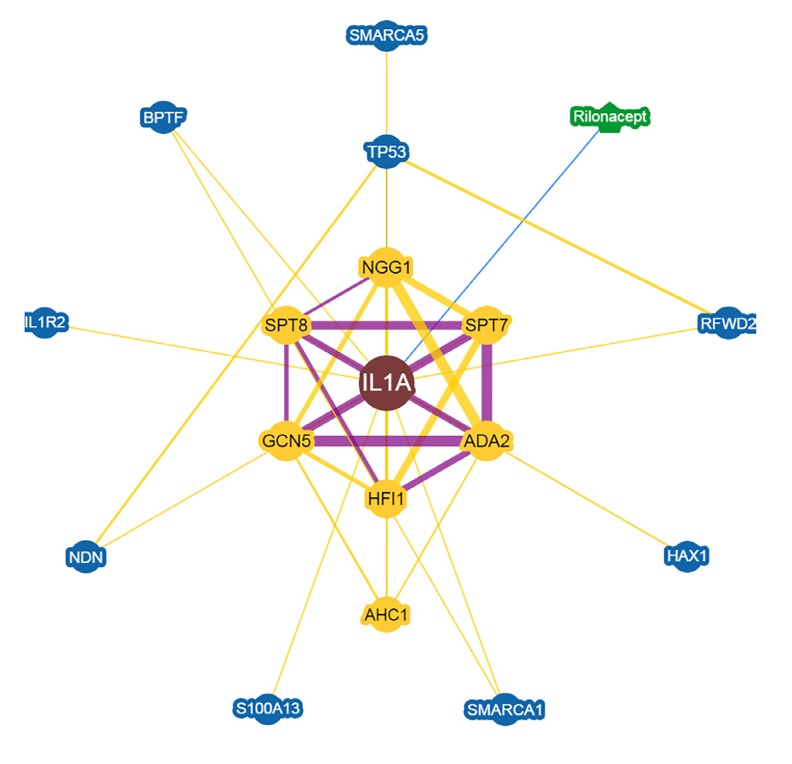
Network of human *IL-1α* interactions based on BioGRID. *IL-1α* interacts with 17 other molecules. Purple and yellow lines show interactions detected by genetic and physical experiments respectively.

Functional consequence of the C376A transversion on RNA structure was evaluated. However, no significant effect on RN (distance: 0.0191, P=0.686) was observed (Fig .2). Minimum free energy of normal RNA was equal to -81.80 kcal/mol but increased to -80.50 kcal/mol for the variant allele. The data from NetGene2 revealed that the C370A SNP alters the *IL-1α* splice site pattern on the direct strand (+ strand) especially for the acceptor splice pattern (Fig .2). The BioGRID interactome showed that *IL-1α* has 17 gene-gene interactions (Fig .3).

**Table 1:** Allelic and genotypic distribution of the *IL-1α C376A* SNP


Genotype/Allele	n (%)					OR (95% CI)				P value			
	Controln=230	All casesn=230	Oligon=95	Astenon=84	NOAn=51	Total	Oligo	Asteno	Azo	Total	Oligo	Asteno	Azo

AA	44(19.13)	27(11.74)	14(14.74)	8(9.52)	5(9.80)	-	-	-	-	-	-	-	-
AC	90(39.13)	87(37.83)	30(31.58)	39(46.43)	18(35.29)	1.58(0.90-2.74)	1.05(0.51-2.17)	2.38(1.03-5.53)	1.76(0.61-5.05)	0.113	0.901	0.043	0.293
CC	96(41.74)	116(50.43)	51(53.68)	37(44.05)	28(54.90)	1.97(1.14-3.41)	1.67(0.84-3.33)	2.12(0.91-4.93)	2.57(0.93-7.09)	0.016	0.146	0.081	0.069
AC+CC	186(80.87)	203(88.26)	81(85.26)	76(90.48)	46(90.20)	1.78(1.06-2.99)	1.37(0.71-2.64)	2.25(1.01-4.10)	2.18(0.82-5.80)	0.030	0.348	0.047	0.127
A	178(38.70)	141(30.65)	58(30.53)	55(32.74)	28(27.45)	-	-	-	-	-	-	-	-
C	282(61.30)	319(69.35)	132(69.47)	113(67.26)	74(72.55)	1.43(1.09-1.88)	1.44(1.01-2.06)	1.30(0.89-1.88)	1.67(1.04-2.68)	0.011	0.049	0.172	0.034


SNP; Single nucleotide polymorphism, OR; Odds ratio, Oligo; Oligozoospermia, Asteno; Asthenozoospermia, and NOA; Non-obstructive azoospermia. Significant differences between the case and control groups are shown in bold type.

## Discussion

In this study, we examined the association of the *IL-1α* C376A SNP with male infertility in an Iranian population (Kashan, Iran) as a pilot study. Our study revealed that not only the CC genotype was associated with male infertility, but also the C allele showed significant association. In addition, carriers of the C allele were at almost two-fold risk for male infertility. Sub-group analysis revealed that AC genotype and carriers of C were associated with asthenozoospermia. Also, the C allele was significantly associated with oligozoospermia and non-obstructive azoospermia. Therefore, *IL-1α* C376A is a potential genetic risk factor for male infertility, although further studies of different ethnicities in Iran and other populations are required to obtain a more accurate picture. After Hardy- Weinberg equilibrium (HWE) calculation in the control group, we found a highly significant deviation. However, the case group showed no deviation. even though it does not necessarily need to follow HWE due to the inherent sampling bias in cases. The deviation from HWE in the control group (normozoospermic men) could also be due to the selection bias (27) given that not all men in the general population will be fertile.

Spermatogenesis is a dynamic process in which many factors are necessary for creating and regulating balance in this process. For example, growth factors and cytokines are essential for development of functional spermatozoa (28, 29). Interleukin-1 is produced by epithelia of seminiferous tubules and acts as a physiological paracrine/ autocrine factor on testicular cells and required for immunological protection (30). There is a probable mechanism that in the absence of testosterone, followed by increased cell apoptosis, spermatogenesis is finally reduced (31, 32). The second probable mechanism is excess reactive oxygen species (ROS).

The presence of the associated SNP and the consequent change in the amount of interleukin along with excess production of ROS may reduce sperm motility. One of the reasons for reduced sperm motility may be DNA damage and lipid peroxidation of sperm membrane (33). Also, increased ROS with oxidizing DNA or proteins, enzyme inhibition, cell death and apoptosis of sperm may cause the oligozoospermia phenotype (34, 35). Due to these possible mechanisms, the association of the *IL-1α* SNP with some abnormalities in sperm parameter may be explained. SNPs could change the gene expression pattern (14), mRNA structure (36, 37), splicing pattern (38) and protein function (39, 40). In silico tools, which can predict the damaging effects of SNPs, were therefore used especially that *IL-1α* C376A is an intronic SNP and may affect RNA structure and splicing. Although we found no evidence for C376A to affect RNA structure, we observed a predicted effect on splicing alteration. Therefore, the association of this SNP may be due to this effect. In this study, there were various limitations including gene-environment and gene-gene interactions that must be considered in subsequent studies. Also, lack of *in vitro* studies such as investigating the effect of the SNP on *IL-1α* gene expression and isoform formations due to splicing alterations is another limitation of this study.

## Conclusion

Our study suggests that the *IL-1α* C376A SNP may increase the risk of male infertility up to two-fold. Since this is the first study, future studies with larger sample sizes in different ethnicities and populations is warranted given the variable environmental factors in different geographic regions.

## References

[1] Agarwal A, Roychoudhury S, Sharma R, Gupta S, Majzoub A, Sabanegh E (2017). Diagnostic application of oxidation-reduction potential assay for measurement of oxidative stress: clinical utility in male factor infertility. Reprod Biomed Online.

[2] Tremellen K (2008). Oxidative stress and male infertility--a clinical perspective. Hum Reprod Update.

[3] Agarwal A, Said TM (2003). Role of sperm chromatin abnormalities and DNA damage in male infertility. Hum Reprod Update.

[4] Poongothai J, Gopenath TS, Manonayaki S (2009). Genetics of human male infertility. Singapore Med J.

[5] Ren Z, Ren P, Yang B, Fang K, Ren S, Liao J (2017). MTHFR C677T, A1298C and MS A2756G gene polymorphisms and male infertility risk in a Chinese population: a meta-analysis. PLoS One.

[6] Talebi E, Karimian M, Nikzad H (2018). Association of sperm mitochondrial DNA deletions with male infertility in an Iranian population. Mitochondrial DNA A DNA Mapp Seq Anal.

[7] Rafatmanesh A, Nikzad H, Ebrahimi A, Karimian M, Zamani T (2018). Association of the c.-9C>T and c.368A>G transitions in H2BFWT gene with male infertility in an Iranian population. Andrologia.

[8] Fraczek M, Kurpisz M (2015). Cytokines in the male reproductive tract and their role in infertility disorders. J Reprod Immunol.

[9] Fraczek M, Kurpisz M (2007). Inflammatory mediators exert toxic effects of oxidative stress on human spermatozoa. J Androl.

[10] Rozwadowska N, Fiszer D, Kurpisz M (2005). Function of the interleukin-1 gene system in immunomodulation, apoptosis and proliferation in the male gonad. Postepy Hig Med Dosw (Online).

[11] Isik N, Arman A, Canturk IA, Gurkan AC, Candan F, Aktan S (2013). Multiple sclerosis: association with the interleukin-1 gene family polymorphisms in the Turkish population. Int J Neurosci.

[12] Wood IS, Wang B, Trayhurn P (2009). IL-33, a recently identified interleukin-1 gene family member, is expressed in human adipocytes. Biochem Biophys Res Commun.

[13] Rozwadowska N, Fiszer D, Jedrzejczak P, Kosicki W, Kurpisz M (2007). Interleukin-1 superfamily genes expression in normal or impaired human spermatogenesis. Genes Immun.

[14] Jamali S, Karimian M, Nikzad H, Aftabi Y (2016). The c.-190 C>A transversion in promoter region of protamine1 gene as a genetic risk factor for idiopathic oligozoospermia. Mol Biol Rep.

[15] Nikzad H, Karimian M, Sareban K, Khoshsokhan M, Hosseinzadeh Colagar A (2015). MTHFR-Ala222Val and male infertility: a study in Iranian men, an updated meta-analysis and an in silico-analysis. Reprod Biomed Online.

[16] Karimian M, Hosseinzadeh Colagar A (2016). Methionine synthase A2756G transition might be a risk factor for male infertility: Evidences from seven case-control studies. Mol Cell Endocrinol.

[17] Charbonneau B, Block MS, Bamlet WR, Vierkant RA, Kalli KR, Fogarty Z (2014). Risk of ovarian cancer and the NF-κB pathway: genetic association with IL1A and TNFSF10. Cancer Res.

[18] Camargo MC, Mera R, Correa P, Peek RM Jr, Fontham ET, Goodman KJ (2006). Interleukin-1beta and interleukin-1 receptor antagonist gene polymorphisms and gastric cancer: a meta-analysis. Cancer Epidemiol Biomarkers Prev.

[19] Kawaguchi Y, Tochimoto A, Ichikawa N, Harigai M, Hara M, Kotake S (2003). Association of IL1A gene polymorphisms with susceptibility to and severity of systemic sclerosis in the Japanese population. Arthritis Rheum.

[20] Yin WT, Pan YP, Lin L (2016). Association between IL-1α rs17561 and IL-1β rs1143634 polymorphisms and periodontitis: a meta-analysis. Genet Mol Res.

[21] Hata Y, Nakaoka H, Yoshihara K, Adachi S, Haino K, Yamaguchi M (2013). A nonsynonymous variant of IL1A is associated with endometriosis in Japanese population. J Hum Genet.

[22] Wang Y, Jin T, Zhang X, Wei W, Cui Y, Geng T (2013). Common single nucleotide polymorphisms and keratoconus in the Han Chinese population. Ophthalmic Genet.

[23] World Health Organisation (1999). WHO laboratory manual for the examination of human semen and sperm-cervical mucus interaction.Cambridge.Cambridge University Press.

[24] Sabarinathan R, Tafer H, Seemann SE, Hofacker IL, Stadler PF, Gorodkin J (2013). The RNAsnp web server: predicting SNP effects on local RNA secondary structure. Nucleic Acids Res.

[25] Hebsgaard SM, Korning PG, Tolstrup N, Engelbrecht J, Rouzé P, Brunak S (1996). Splice site prediction in Arabidopsis thaliana pre-mRNA by combining local and global sequence information. Nucleic Acids Res.

[26] Chatr-Aryamontri A, Breitkreutz BJ, Oughtred R, Boucher L, Heinicke S, Chen D (2015). The BioGRID interaction database: 2015 update. Nucleic Acids Res.

[27] Namipashaki A, Razaghi-Moghadam Z, Ansari-Pour N (2015). The essentiality of reporting Hardy-Weinberg equilibrium calculations in population-based genetic association studies. Cell J.

[28] Diemer T, Hales DB, Weidner W (2003). Immune-endocrine interactions and Leydig cell function: the role of cytokines. Andrologia.

[29] Jaiswal D, Trivedi S, Singh R, Dada R, Singh K (2012). Association of the IL1RN gene VNTR polymorphism with human male infertility. PLoS One.

[30] Huleihel M, Lunenfeld E (2004). Regulation of spermatogenesis by paracrine/autocrine testicular factors. Asian J Androl.

[31] Mfuna Endam L, Cormier C, Bossé Y, Filali-Mouhim A, Desrosiers M (2010). Association of IL1A, IL1B, and TNF gene polymorphisms with chronic rhinosinusitis with and without nasal polyposis: a replication study. Arch Otolaryngol Head Neck Surg.

[32] Engels EA, Wu X, Gu J, Dong Q, Liu J, Spitz MR (2007). Systematic evaluation of genetic variants in the inflammation pathway and risk of lung cancer. Cancer Res.

[33] Martínez P, Proverbio F, Camejo MI (2007). Sperm lipid peroxidation and pro-inflammatory cytokines. Asian J Androl.

[34] Agarwal A, Virk G, Ong C, du Plessis SS (2014). Effect of oxidative stress on male reproduction. World J Mens Health.

[35] Aprioku JS (2013). Pharmacology of free radicals and the impact of reactive oxygen species on the testis. J Reprod Infertil.

[36] Mazaheri M, Karimian M, Behjati M, Raygan F, Hosseinzadeh Colagar A (2017). Association analysis of rs1049255 and rs4673 transitions in p22phox gene with coronary artery disease: a case-control study and a computational analysis. Ir J Med Sci.

[37] Zamani-Badi T, Karimian M, Azami-Tameh A, Nikzad H (2017). Association of C3953T transition in interleukin 1â gene with idiopathic male infertility in an Iranian population.Hum Fertil (Camb).

[38] Soleimani Z, Kheirkhah D, Sharif MR, Sharif A, Karimian M, Aftabi Y (2017). Association of CCND1 gene c.870G> A polymorphism with breast cancer risk: a case-control study and a meta-analysis. Pathol Oncol Res.

[39] Ebrahimi A, Hosseinzadeh Colagar A, Karimian M (2017). Association of human methionine Synthase-A2756G transition with prostate cancer: a case-control study and in Silico analysis. Acta Med Iran.

[40] Raygan F, Karimian M, Rezaeian A, Bahmani B, Behjati M (2016). Angiotensinogen-M235T as a risk factor for myocardial infarction in Asian populations: a genetic association study and a bioinformatics approach. Croat Med J.

